# Determinants of circumcision and willingness to be circumcised by Rwandan men, 2010

**DOI:** 10.1186/1471-2458-12-134

**Published:** 2012-02-18

**Authors:** Rwego A Gasasira, Malabika Sarker, Landry Tsague, Sabin Nsanzimana, Aimée Gwiza, Jennifer Mbabazi, Corine Karema, Anita Asiimwe, Placidie Mugwaneza

**Affiliations:** 1 Institute of Public Health, University of Heidelberg, Heidelberg, Germany; 2 UNICEF Rwanda, Kigali, Rwanda; 3 Rwanda Biomedical Center/Institute of HIV/AIDS, Disease Prevention and Control (RBC/IHDPC), Kigali, Rwanda

**Keywords:** Male Circumcision, HIV/AIDS, HIV Prevention, Rwanda

## Abstract

**Background:**

Male Circumcision (MC) has been recommended as one of the preventive measures against sexual HIV transmission by the World Health Organization (WHO). Rwanda has adopted MC as recommended but the country is a non-traditionally circumcising society. The objective was to explore knowledge and perception of Rwandan men on Male Circumcision (MC) and to determine the factors associated with the willingness to be circumcised and to circumcise their sons.

**Methods:**

This cross sectional study was conducted in 29 districts of Rwanda between January and March 2010. Data were collected using a structured questionnaire among men aged 15-59 years. The rate of MC was measured and its perception from respondents, and then the factors associated with the willingness to go for MC were analysed using multiple logistic regressions.

**Results:**

A total of 1098 men were interviewed. Among respondents 17% (95% CI 14-19%) reported being circumcised. About three-quarter (72%) could define MC, but 37% of adolescent could not. Half of the participants were willing to get circumcised and 79% of men would accept circumcision for their sons. The main motivators for MC were its benefits in HIV/STI prevention (69%) and improving hygiene (49%). Being too old was the main reason (32%) reported by men reluctant to undergo MC and younger men were afraid of pain in particular those less than 19 years old (42%). The willingness to circumcise was significantly associated with younger age, living in the Eastern Province, marital status, and the knowledge of the preventive role of circumcision.

**Conclusions:**

Adolescents and young adults were more willing to be circumcised. It is critical to ensure the availability of pain free services in order to satisfy the increasing demand for the scale up of MC in Rwanda.

## Background

Male Circumcision (MC) has been recommended as one of the preventive measures against sexual HIV transmission in 2007 by the World Health Organization (WHO) and UNAIDS [1]. Three Randomized Controlled Trials (RCT) conducted in South Africa, Uganda and Kenya, strongly supported the efficacy of MC at reducing the risk of HIV transmission from infected women to circumcised men, by approximately 60% [2-5]. Studies also reported a substantially reduced risk of other Sexually Transmitted Infections (STIs) such as syphilis, chancroid, and Herpes Simplex-2 (HSV) in circumcised men [6]. The MC procedure is cost effective particularly in adolescent and infant MC [7]. WHO has advocated countries with a generalized HIV epidemic and with a male circumcision rate below 20% to adopting extensively MC for the benefit of the whole population [1].

A recent review from 13 African countries reported that on average 65% of uncircumcised men were willing to get circumcised and 71% were willing to have a son circumcised, although MC was not a common practice in these communities [8]. Higher odds of being circumcised among adults were associated with improved hygiene and a reduced risk of STIs. Male circumcision is associated with four major determinants classified as: Religious, Cultural, Social and Medical [1]. Rwanda being a non-traditionally circumcising society, circumcision is most commonly viewed as a Muslim practice [9].

Rwanda has a generalized HIV epidemic, with 3% HIV prevalence in the general population, yet prevalence of MC among men aged 15-59 years was estimated at 9% in 2005 and 12% in 2008 [9,10]. Unlike Muslims who practice MC (with MC prevalence of 82.4%) [9] as part of religious ritual, the majority of Rwandan who are Christian (93%) does not traditionally practice MC (the prevalence is 9.7% in Catholics, 11.4% in protestants and 12% in Adventists) [9]. In late 2007, the government of Rwanda endorsed MC as an additional preventive method as part of the national strategy to halving the incidence of HIV in the general population by 2012 [11]. Yet, there is knowledge gap with regard to the perception and willingness to undergo male circumcision among Rwandan non circumcised adults and their male children.

This study aimed at filling this gap, and findings will contribute to the development of evidence-based policy and strategies for implementing MC for HIV prevention in Rwanda. The analysis focused on how uncircumcised young men perceived MC compared to older men and the factors influencing their willingness to go for circumcision.

## Methods

The analysis used data from a nationwide survey on knowledge, attitudes and practices regarding MC in general population (MC-KAP) conducted in Rwanda between January and March 2010 by the Center for Treatment and Research on AIDS, Malaria, Tuberculosis and other Epidemics (TRAC Plus)/Rwanda Ministry of Health. The MC-KAP survey included both men and women aged 15-59 for men and 15-49 for women. The main objective of MC KAP survey was to serve as a situational analysis, and guide medium and long term strategic program planning using MC as a preventive method for HIV.

### Sampling

**Figure 1:**
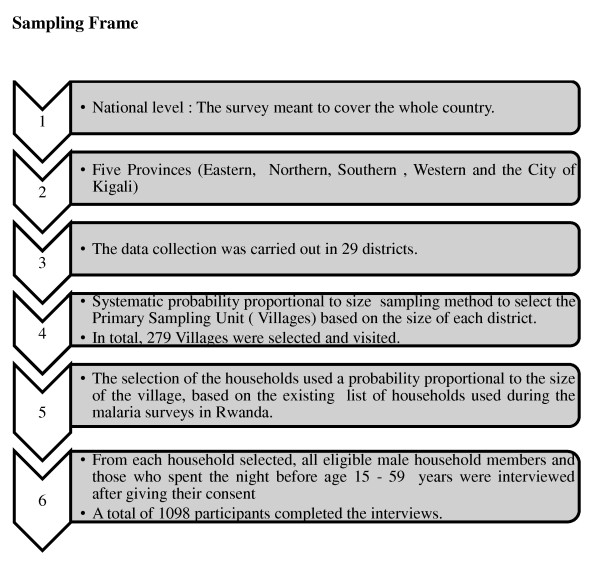
**Sampling frame**.

The MC KAP surveyed a nationally representative sample of 1452 households selected through a two-stage cluster. The sample was first stratified in order to ensure a good representation of all 5 provinces (City of Kigali, Southern, Western, Northern and Eastern Provinces) and all 30 districts of the country. The primary sampling units (PSUs) were the villages called *"imidugudu" *and the second stage included the households. The selection of PSUs was based on the existing list of villages and households, which was constructed based on the updated list of enumeration areas covered in 2002 General Population and Housing Census (RGPH) prepared by the National Institute of Statistics of Rwanda (NISR) [12] (Figure 1).

A systematic probability proportional to size (PPS) sampling method was used to select the PSU in each district. Due to the sampling oversight, the district of Nyanza was not selected, bringing the number of districts covered by the survey to 29. In total, 279 PSUs were selected for the data collection (18% in urban area and 82% in rural area). Within each selected village, households were selected with a probability proportional to the size of the village from the existing list of households used during the malaria indicator survey carried out in 2007 and 2008 in Rwanda along with the interim demographic health survey [9]. Within each selected household, all males aged 15-59 years who consented and had spent the last night in the household were eligible for the study. Assuming that each household had at least one eligible adult male, the estimated number of participants was estimated at 1,452.

### Data collection and data entry

The study questionnaire was developed in English and then translated into Kinyarwanda, (the national language in Rwanda) and pre-tested. Study interviewers were selected and trained during one day on questionnaire administration and other study procedures. All participants were asked to provide signed informed consent prior to interview.

All data were double entered in the statistical package Epi Info™ Version 6.04 and comparison was done and any discrepancies verified.

### Data analysis

The analysis focused on the subset of male who participated in the MC KAP survey, to measure the rate of MC in the Rwandan male population who do not traditionally practice MC. The three main outcome variables were: 1) Circumcision status ("Are you circumcised yourself?"); 2) Willingness to be circumcised ("Would you consider being circumcised?"), and 3) Willingness to have one's son circumcised ("If you have a son, would you circumcise him?").

Univariate analysis and multiple logistic regressions were performed using SAS version 9.2. For logistic regression, the factors that were found to be significant in the univariate analysis were first included into the full model with all potentially important co-variates to adjust for confounding. A step wise regression model was conducted. Variables with no effect in the adjusted model were removed one by one to obtain more robust results [13].

### Ethical considerations

The ethical approval was obtained from Rwanda National Ethics Committee (RNEC) functioning under the Ministry of Health, Rwanda. A written informed consent was obtained from all the study participants after describing to them all the issues related to the study in details.

## Results

### Background characteristics of the study population

The questionnaire in the study was administrated to 1098 men. Table 1 presents the socio-demographic characteristics of the study population by provinces. The distribution of the study population by province was as follows: The City of Kigali with 101 (9.2%), Southern Province with 290 (26%), Western Province with 294 (27%), Northern Province with 214 (20%) and Eastern Province with 199 (18%).

**Table 1:** Socio demographic characteristics of the study participants by Province of Rwanda, 2010 (N = 1096)

	Province					
**Socio demographic characters**	**City of Kigali**	**Southern**	**Western**	**Northern**	**Eastern**	**Total**

Age group	n = 99 (%)	n = 290 (%)	n = 294 (%)	n = 212 (%)	n = 199 (%)	n = 1094 (%)

≤ 19	7 (7)	21 (7.)	57 (19)	34 (16)	27 (14)	146 (14)

20-29	21 (21)	87 (30)	83 (28)	63 (30)	59 (30)	313 (29)

30-39	41 (41)	82 (28)	71 (24)	51 (24)	43 (22)	288 (26)

40+	30 (30)	100 (34)	83 (28)	64 (30)	70 (35)	347 (32)

**Religion**	n = 101 (%)	n = 290 (%)	n = 293 (%)	n = 212 (%)	n = 199 (%)	n = 1095 (%)

Catholic	36 (36)	170 (59)	99 (34)	137 (65)	97 (49)	539 (49)

Protestant	39 (39)	89 (31)	139 (47)	57 (27)	73 (37)	397 (36)

Adventist	10 (10)	27 (9)	46 (16)	10 (5)	20 (10)	113 (10)

Muslim	13 (13)	2 (0.7)	2 (0.7)	6 (3)	8 (4)	31 (3)

Other	3 (3)	2 (0.7)	7 (2)	2 (0.9)	1 (0.5)	15 (1)

**Education**	n = 101 (%)	n = 290 (%)	n = 294 (%)	n = 212 (%)	n = 199 (%)	n = 1096 (%)

Attended school	92 (91)	227 (78)	255 (87)	174 (82)	170 (85)	918 (84)

Never attended school	9 (9)	63 (22)	39 (13)	38 (18)	29 (15)	178 (16)

**Level of education**	n = 101 (%)	n = 290 (%)	n = 294 (%)	n = 212 (%)	n = 199 (%)	n = 1096 (%)

Never attended school	9 (9)	63 (22)	39 (13)	38 (18)	29 (15)	178 (16)

Primary school	45 (49)	194 (86)	206 (81)	154 (89)	135 (79)	734 (80)

Vocational school	11 (12)	9 (4)	12 (5)	4 (2)	4 (2)	40 (4)

Secondary school	25 (27)	24 (11)	33 (13)	15 (9)	25 (15)	122 (13)

University	11 (12)	0 (0.0)	4 (2)	1 (0.6)	6 (4)	22 (2)

**Marital Status**	n = 101 (%)	n = 290 (%)	n = 294 (%)	n = 212 (%)	n = 199 (%)	n = 1096 (%)

Married	37 (37)	162 (56)	151 (51)	103 (49)	88 (44)	541 (49)

Cohabitation	22 (22)	55 (19)	32 (11)	34 (16)	51 (26)	194 (18)

Singles/living alone	42 (42)	73 (25)	111 (38)	75 (35)	60 (30)	361 (33)

Mean age was 33.6 years [standard deviation (sd) = .12 years]. Most (96%) respondents were Christians (49% Catholic; 36% Protestant; and 10% Adventist) followed by Muslim (2%). The majority (84%) of respondents had ever attended school and this proportion was higher in the City of Kigali (91%) (Table 1).

### Prevalence of male circumcision

The prevalence of MC was estimated at 17% [95% Confidence interval (CI): 14%-19%] in the general population, varying from 100% among Muslim community to 14% among non Muslim. The MC prevalence was 13%, 17%, 15%, and 13% of those in the age groups of > 39, 30-39, 20-29 and less than 19 years. Adolescent are 66% less likely to be circumcised compared to adults aged 40 and above [Adjusted Odd Ratio (aOR): 0.34; (95% CI: 0.13, 0.85) (P ≤ 0.05)]. The City of Kigali had the highest prevalence with 53% and the Southern province had the lowest (4%). Compared to men in Kigali, men in the North province had the lowest likelihood to be circumcised [aOR: 0.09; (95% CI: 0.04, 0.22) (P ≤ 0.001)]. Men who lived in urban settings (44%) were more circumcised than those who lived in rural areas (10%). There was no significant difference of MC prevalence among non Muslims. The MC prevalence was 10%, 20% and 11% among Catholics, Protestants and Adventists respectively. Eighteen percent of the men who attended school were circumcised while only 9% of the men who never attended school were found to be circumcised. Prevalence of circumcision was found to be higher in men who attended universities and secondary schools with 82% and 41% respectively. Single or living alone men were more circumcised (23%) compared to married men (15%). Within provinces, there are disparities among districts. Rusizi had the highest prevalence of male circumcision (71%) followed by the three districts of Kigali city: Kicukiro (56%), Gasabo (56%) and Nyarugenge (49%). None interviewed in the districts of Nyabihu (West), Kayonza (East) and Kamonyi (South) were circumcised.

### Knowledge/awareness of Male circumcision

In general, circumcision was well known among men who participated in the study, 72% of the respondents correctly defined circumcision as 'removal of the entire foreskin'. While all Muslims participating in the study (n = 31) defined MC correctly, 52% of them defined it as partial removal of the foreskin. Among non Muslims, 31% (n = 535) of Catholics were not able to define MC. Thirty seven percent of adolescents (≤ 19 years) could not define correctly MC.

### Perception /Attitudes of uncircumcised men (UCM) towards circumcision

Half of the interviewed UCM were willing to get circumcised, with the highest demand from Eastern province (62%). The main motivators for MC among UCM were its benefits for preventing sexually transmitted Infections (STI) including HIV (69%) and improving hygiene (49%). Amongst the men belonging to the age group 20-29 years, 74% (n = 312) mentioned STI/HIV prevention as the main reason for MC. The predominant reason for not to be circumcised was being too old (32%). Forty-eight men (11%) mentioned that circumcision was not needed because they were not sexually promiscuous. While the men belonging to the age groups above 29 years mostly did not want to be circumcised because of older age, younger were afraid of pain, particularly those less than 19 years old (42%).

Married men (60%) and those who were in cohabitation (49%) were not interested in circumcision. After informing the respondents that studies have shown that circumcision done by trained professionals reduces the risk of HIV infection by 60%, majority of the men supported their son's MC (79%), and 89% of them preferred to do it at younger age (below 15 years).

### Determinants of circumcision among non Muslim men

In uni variate analysis, people living in four provinces were less likely to be circumcised (OR: = 0.04, 0.41, 0.05, 0.12) compared to the City of Kigali. The men in cohabitation (OR: = 0.34) were also less likely to be circumcised compared to those who were married. Significant association was found with men having secondary education (OR=: 8), University (OR=: 58) education, and being Protestants (OR=: 2.18).

With other factors controlled, being circumcised was significantly associated with men who had either secondary (aOR=: 4), or university education (aOR=: 17), and those who mentioned hygiene as a reason to go for circumcision (aOR=: 4). Other associated factors were living in Southern (aOR=: 0.10), Northern (aOR = 0.09), or Eastern (aOR = 0.17) province, and men who were in cohabitation (aOR=: 0.35) (Table 2).

**Table 2:** Determinants of circumcision among non Muslim men, 2010

Variable	Crude OR (95% CI)	Adjusted OR (95% CI)
**Age group**		

40+	1.00	1.00

30-39	1.43 (0.91, 2.26)	0.82 (0.46, 1.45)

20-29	1.22 (0.77, 1.93)	0.67 (0.34, 1.31)

≤ 19	1.11 (0.62, 1.98)	0.34 (0.13, 0.85)*

**Education**		

No education	1.00	1.00

Primary and vocational	1.61 (0.86, 3.03)	1.26 (0.62, 2.57)

Secondary	7.73 (3.84, 15.58)***	4.01 (1.77, 9.39)***

University	57.73 (16.76, 198.88)***	17.07 (4.06, 71.82)***

**Religion**		

Catholic	1.00	1.00

Protestant	2.18 (1.5, 3.16)***	1.54 (0.97, 2.44)

Adventist	1.04 (0.54, 2.02)	0.69 (0.32, 1.47)

Others	3.19 (0.98, 10.35)*	2.05 (0.45, 9.30)

**Province**		

City of Kigali	1.00	1.00

Southern	0.04 (0.02, 0.09)***	0.10 (0.04, 0.23)***

Western	0.41 (0.25, 0.67)***	0.08 (0.43, 1.47)

Northern	0.05 (0.02, 0.12)***	0.09 (0.04, 0.22)***

Eastern	0.12 (0.06, 0.23)***	0.17 (0.08, 0.37)***

**Marital Status**		

Married	1.00	1.00

Cohabiting	0.34 (0.17, 0.68)**	0.35 (0.16, 0.76)**

Single/Living alone	1.42 (0.98, 2.04)	1.72 (0.89, 3.35)

**Reasons to circumcise**		

STI/HIV (No)	1.00	1.00

STI/HIV (Yes)	2.18 (1.41, 3.37)***	1.65 (0.99, 2.76)

Hygiene (No)	1.00	1.00

Hygiene (Yes)	5.08 (3.39, 7.61)***	4.18 (2.63, 6.63)***

Significant at *P≤0.05, **P≤0.01, ***P≤0.001

### Factors associated with the willingness to circumcise amongst UCM and their support to circumcise their son/s

In the uni variate analysis, it was found that younger men (30-39, 20-29, < = 19 years) were more likely to get circumcised compared to the older ones (OR = 2.89, 4.36, 4.76), as well as education, living in Eastern Province, marital status, MC knowledge, and preventive role of MC also are significantly associated with the willingness to circumcise. Controlling for other factors, except education, all variables mentioned above found to be significantly associated with willingness to circumcise (Table 3).

**Table 3:** Determinants of willingness to circumcise among non Muslim UCM and their support to circumcise their sons, 2010

	Determinants of willingness to circumcise		Determinants of the support for Son's MC	
**Variable**	**Crude OR (95% CI)**	**Adjusted OR (95% CI)**	**Crude OR (95% CI)**	**Adjusted OR (95% CI)**

Age group				

40+	1.00	1.00	1.00	1.00

30-39	2.89 (2.01, 4.14)***	3.04 (2.06, 4.48)***	1.18 (0.78, 1.79)	1.07 (0.68, 1.69)

20-29	4.36 (3.05, 6.24)***	3.11 (2.03, 4.76)***	1.83 (1.18, 2.82)	1.62 (0.94, 2.78)

≤19	4.76 (3.04, 7.47)***	2.90 (1.52, 5.56)***	1.05 (0.64, 1.72)	1.47 (0.68, 3.18)

**Education**				

No education	1.00	1.00	1.00	1.00

Primary and vocational	1.41 (1.00, 2.00)*	0.96 (0.64, 1.44)	1.24 (0.83, 1.87)	0.89 (0.56, 1.42)

Second, and University	2.51 (1.43, 4.41)**	0.99 (0.51, 1.90)	2.17 (1.02, 4.64)*	0.92 (0.39, 2.16)

**Religion**				

Catholics	1.00	1.00	1.00	1.00

Protestants	1.34 (1.01, 1.78)*	1.14 (0.82, 1.58)	0.81 (0.57, 1.15)	0.71 (0.48, 1.05)

Adventists	1.07 (0.69, 1.64)	0.82 (0.50, 1.34)	0.78 (0.47, 1.30)	0.53 (0.29, 0.94)*

Others	1.33 (0.40, 4.42)	2.41 (0.60, 9.72)	1.11 (0.24, 5.22)	1.93 (0.35, 10.62)

**Province**				

City of Kigali	1.00	1.00	1.00	1.00

Southern	1.10 (0.59, 2.05)	1.61 (0.80, 3.26)	0.91 (0.43, 1.94)	1.27 (0.54, 2.96)

Western	1.48 (0.79, 2.77)	1.81 (0.88, 3.73)	1.04 (0.48, 2.25)	1.56 (0.65, 3.72)

Northern	0.98 (0.52, 1.84)	1.03 (0.50, 2.13)	0.71 (0.33, 1.53)	0.83 (0.35, 1.98)

Eastern	2.10 (1.10, 4.02)*	2.93 (1.40, 6.13)*	1.72 (0.75, 3.92)	2.23 (089, 5.57)

**Marital Status**				

Married	1.00	1.00	1.00	1.00

Cohabiting	1.55 (1.09, 2.19)*	1.30 (0.87, 1.93)	1.30 (0.84, 2.03)	1.20 (0.73, 1.98)

Singles/Living alone	3.06 (2.24, 4.18)***	2.40 (1.50, 3.85)*	1.11 (0.76, 1.60)	0.86 (0.46, 1.54)

**Knowledge of MC**				

Don't know	1.00	1.00	1.00	1.00

Removal of the entire foreskin	2.16 (1.53, 3.05)***	1.50 (1.01, 2.24)*	2.64 (1.74, 4.00)***	1.63 (1.02, 2.60)*

Removal of the foreskin partially	2.26 (1.65, 3.09)***	1.59 (1.09, 2.31)*	4.05 (2.72, 6.04)***	2.46 (1.56, 3.86)***

**Reasons to circumcise**				

STI (No)	1.00	1.00	1.00	1.00

STI (Yes)	2.68 (2.01, 3.57)***	2.12 (1.52, 2.95)***	4.00 (2.85, 5.59)***	2.82 (1.95, 4.09)***

Hygiene (No)	1.00	1.00	1.00	1.00

Hygiene (Yes)	2.21 (1.68, 2.89)***	2.20 (1.60, 3.03)***	3.01 (2.07, 4.39)***	2.20 (1.45, 3.34)***

Significant at *P≤0.05, **P≤0.01, ***P≤0.001

However, only MC knowledge (aOR = 1.63, 2.46), the role of MC on STI prevention (aOR = 2.82), and improved hygiene (aOR = 2.20), were significantly associated with the willingness to get their sons circumcised. On the other hand, Adventists were less likely to circumcise their son/s compared to the Catholics (aOR: = 0.53) (Table 3).

## Discussion

This community based study presents the determinants of circumcision and willingness to circumcision among men in Rwanda. The overall prevalence of circumcision was 17% among study population but varied from provinces and districts. The city of Kigali has the highest prevalence (52%) followed by the Western province. There is an increase in national and provincial circumcision rate compared to previous two surveys conducted in Rwanda, the Demographic Health Survey 2005 (9%) and the Intermediate Demographic Health Survey 2008 (12%). However, in 2008 survey, the city of Kigali (35.3%) and the Western Province (18%) had the highest prevalence compared to other provinces [9,10].

The reason for high acceptance rate of circumcision in the City of Kigali is because of the coupling effect of access to information and education compared to other provinces. After the publication of the studies carried out in Kenya, Uganda and South Africa concluding that male circumcision can prevent heterosexual HIV infection from female to male up to 60% [2-4], both print and visual media in Kigali focused on the benefits of MC. This is also similar to Uganda, where the capital city of Kampala has the highest prevalence of MC compared to the other regions in the country [14]. The spread of Muslim religion in Kigali could be another explanation where young men are mixing in schools, sports and social events. This effect was evident in Tanzania where circumcision was found to increase in the ethnic groups (who are traditionally not used to circumcising) because of contacts with circumcising groups especially in schools and other social mixing [15]. The district of Rusizi in the Western Province sharing the border with The Democratic Republic of Congo (DRC), had the highest prevalence of circumcision (71%). The close contact and exchange with people in DRC, a country where circumcision is a common practice (97%) [16] could be a strong explanation of such high prevalence. Although after controlling for other variables place for living was not significant anymore.

Many participants defined circumcision as a partial removal of the foreskin, including Muslims. This self reported information proclaims the existence of different styles of circumcision practiced in the community. Several authors in Africa reported three categories of circumcision: "not circumcised: foreskin completely covered the glans of penis; partially circumcised: foreskin partly covered the glans; completely circumcised: foreskin did not cover the glans at all" [17,18]. Presence of these categories raises concern over two major issues: the training of Health Care providers to perform effective circumcision and the role of the remaining foreskin to increase or to reduce the risk of HIV transmission from female to male.

Higher education was significantly associated with being circumcised. This corroborates with other African countries like Kenya, Ethiopia, Tanzania and Uganda [1]. Knowledge on STI/HIV prevention had a significant positive effect not only in men who were circumcised but also in UCM. The prevention of STI and improved hygiene is similar to the findings of other studies as factors associated with the acceptability of MC [8]; [19]. In Zambia, most of the participants reported that if MC is proven to reduce risk for HIV and STIs, they would seek circumcision for themselves or their sons [20]. In South Africa, more than 70% of UCM report that they would want to be circumcised if MC were effective to protect against STIs [21].

The half of the participants (50.2%) in this study was willing to circumcise and 78.5% considered their son/s to be circumcised. These findings are similar to those found in other African communities where circumcision was not practiced traditionally. A review of studies carried out in Botswana, Kenya, Malawi, South Africa, Swaziland, Uganda, Tanzania, Zambia and Zimbabwe, showed that the median proportion of men willing to circumcise was 65% with a range between 29% in Uganda and 87% in Swaziland. In addition, 71% supported the circumcision of their male child/children [8]. In another study in Dominican Republic, 29% of men were willing to go for circumcision [19]. Those who are young (below 25 years) are more willing to circumcise than the older ones. Similar findings were reported in Dominican Republic and in some African studies where older men are more agreeable to the procedure for their children rather than themselves [8]; [19].

Challenges remain in expanding access to circumcision and addressing cultural concerns about the acceptability of the intervention [22]. The promising fact of this study was that overall willingness for self circumcision and son/s to be circumcised was high. Young people in this study were willing to go for circumcision but fear of pain was found to be a major concern. In other African studies, the major barriers to the acceptability of MC were the fear of pain, concerns for safety and the cost of the procedure [8]. A recent report from Rwanda argued that while adolescent MC is highly cost effective, adult MC is neither cost-saving nor highly cost effective when only the direct benefit for the circumcised man is considered [7].

World Health Organisation recommends that setting with high prevalence, generalized or hyper endemic heterosexual HIV epidemics and low circumcision rates should consider increasing access to circumcision as an additional HIV prevention strategy [23]. On the other hand, concerns have been expressed over inflated sense of STI/HIV protection of circumcised men which can promote high risk behavior [3,24-27]. Nevertheless, adequate counseling on risk reduction can inhibit the adverse behavioral pattern. Similarly engaging in sex during healing period after circumcision may cause increased exposure to infection but appropriate counseling on the abstinence period could potentially reduce such behavior [28].

The study has several strengths. In this nationwide survey participants were nationally distributed, all five provinces and 29 out of 30 districts were covered. The high participation rate (75.6%) and bigger sample size is an indicator for national representation and thus results can be generalized. On the other hand, few limitations are needed to be acknowledged. Firstly self reported circumcision status because the validity of self reported answer without direct observation was questionable. Secondly the quantitative nature of the study didn't allow in depth exploration of knowledge and perceptions as well as attitudes of participants towards circumcision.

## Conclusions

The results showed that adolescents and young adults were more willing to be circumcised. Education, access to information on role of MC on STI/HIV prevention, and elimination of fear associated with circumcision are pivotal to increase the acceptability and uptake of MC among men in Rwanda. In this study, 9.4% of circumcisions were performed by traditional practitioner. It is critical to ensure the availability of services in health facilities and trained providers, in order to satisfy the possible increasing demand of MC. However setting up a system for routinely offering circumcision with safe procedure is a formidable challenge as health system requires both skilled resources and funding [22]. In countries with low prevalence of MC, strategies should include the use of a variety of communication tools to improve knowledge about MC to a wide audience, trainings of providers on safe circumcision and adopt a variety of culturally appropriate options including scaling up neonatal MC, to increase the coverage. Finally, it may take years before the necessary human trial of an effective treatment against HIV is possible [29]. Therefore synergies between preventive and biological intervention like male circumcision can confer greater benefit to men and thus contribute indirect benefit on women too.

## Competing interests

The authors declare that they have no competing interests.

## Authors' contributions

RAG, PM, AA, SN and CK contributed to the design of the program and the study protocol. PM, AG and JM supervised the data collection process. RAG and MS conducted data analysis and interpretation and wrote the first draft of the manuscript. LT reviewed the data analysis and the discussion section. All the authors reviewed and approved the final manuscript.

## Pre-publication history

The pre-publication history for this paper can be accessed here:

http://www.biomedcentral.com/1471-2458/12/134/prepub
